# Robust RNA-Seq of aRNA-amplified single cell material collected by patch clamp

**DOI:** 10.1038/s41598-020-58715-y

**Published:** 2020-02-06

**Authors:** Jae Mun “Hugo” Kim, Adrian Camarena, Christopher Walker, Ming Yi Lin, Victoria Wolseley, Tade Souaiaia, Matthew Thornton, Brendan Grubbs, Robert H. Chow, Oleg V. Evgrafov, James A. Knowles

**Affiliations:** 1 Zhilka Neurogenetic institute, University of Southern California, 1501 San Pablo St, Los Angeles, CA 90033 USA; 2 SUNY Downstate Medical Center 450 Clarkson Ave, Brooklyn, NY 11203 USA; 3 University of California, San Diego 9500 Gilman Dr, La Jolla, CA 92093 USA; 4 University of Chicago, Pritzker School of Medicine 924 E 57th St Suite 104, Chicago, IL 60637 USA

**Keywords:** Next-generation sequencing, Cellular neuroscience

## Abstract

Most single cell RNA sequencing protocols start with single cells dispersed from intact tissue. High-throughput processing of the separated cells is enabled using microfluidics platforms. However, dissociation of tissue results in loss of information about cell location and morphology and potentially alters the transcriptome. An alternative approach for collecting RNA from single cells is to re-purpose the electrophysiological technique of patch clamp recording. A hollow patch pipette is attached to individual cells, enabling the recording of electrical activity, after which the cytoplasm may be extracted for single cell RNA-Seq (“Patch-Seq”). Since the tissue is not disaggregated, the location of cells is readily determined, and the morphology of the cells is maintained, making possible the correlation of single cell transcriptomes with cell location, morphology and electrophysiology. Recent Patch-Seq studies utilizes PCR amplification to increase amount of nucleic acid material to the level required for current sequencing technologies. PCR is prone to create biased libraries – especially with the extremely high degrees of exponential amplification required for single cell amounts of RNA. We compared a PCR-based approach with linear amplifications and demonstrate that aRNA amplification (*in vitro* transcription, IVT) is more sensitive and robust for single cell RNA collected by a patch clamp pipette.

## Introduction

Neuronal cell types are commonly defined by their location, distinct morphology, unique electrophysiology, and expression of specific protein markers. The introduction of RNA sequencing (RNA-Seq) expanded the toolset for characterization of neurons; however, the original RNA-Seq techniques were not able to perform expression profiling of individual cells, limiting our ability to investigate cellular heterogeneity. We^1^, as well as other groups^2–5^, have developed protocols with sufficient sensitivity to perform transcriptome profiling at the single cell level.

Most of such approaches involve tissue dissociation to produce a single cell suspension, followed by sorting of the cells by fluorescence-activated cell sorting (FACS)^6,7^, or using microfluidic devices such as those employed in Drop-Seq^5,8^ or the Chromium controller (10x Genomics)^9^. Unfortunately, the treatments used to dissociate tissues lead to loss of complex morphology of individual neurons, eliminate location information, destroy cell connectivity, and irreversibly change the electrophysiological properties of neurons. Furthermore, dissociation may trigger changes in the transcription profile of cells^4,10,11^.

Patch clamp was invented to record cellular electrical activity, which is often specific to a given cell or neuron type; however, whether or not electrophysiological recording is performed, the patch clamp pipettes can be used to collect individual cells or their contents from within intact or sliced tissue. Cells may be chosen for study based on their morphology, electrophysiological properties and/or location in the tissue or organ. We developed a protocol using patch clamp for collection of cellular contents followed by RNA-Seq^1^. This approach has been used by others (Patch-Seq)^12^, utilizing the SMARTer technique^13^, in which PCR amplification is used to produce sufficient amounts of DNA for sequencing.

Whole transcriptome sequencing of single cell material requires amplification of minute amounts of RNA/cDNA – typically in the range of 10 pg, or even less. If PCR amplification is employed and the number of thermal cycles is large, biased amplification of certain RNA species becomes noticeable, for example, favoring cDNA fragments of shorter length or of lower GC content^14,15^. These challenges led us to investigate alternative linear amplifications methods of DNA or RNA, which are less prone to such bias^16^. Two major approaches are available for linear amplification of nucleic acids: (a) isothermal DNA amplification, which is implemented in Ovation® RNA-Seq System V2 (NuGEN, #7102) kits, and (b) aRNA amplification, which utilizes *in vitro* transcription (IVT)^17^.

Support for the utility of linear amplification methods for single cell analysis came from an analysis using ERCC (External RNA Controls Consortium) spike-in controls, which showed that aRNA amplification used for single cell transcriptome applications, such as the CEL-Seq^8^ and CEL2^18^ protocols, outperformed the PCR-based protocols. CEL-Seq and CEL2 require multiplexing, involving use of barcoded primers to enable parallel processing steps. However, the benefits of multiplexing diminish, and complexity of the protocol becomes an unnecessary complication with lower numbers of samples, such as for electrophysiological patch clamp measurements, when every single cell is collected through a lengthy process.

In this study we focused on protocols that can be applied to single cell or sub-single cell material and that do not require multiplexing. We chose the Ovation® RNA-Seq System V2 kit for linear DNA amplification, and the aRNA method for linear RNA amplification. The NuGEN kit was originally designed for 500 pg input RNA (the amount of RNA in ~50 cells), thus we modified the protocol to work with single cell RNA amounts^19^.

The aRNA method uses *in vitro* transcription of cDNAs for linear RNA amplification, as first described in 1992^20^. Several modifications of this technique had been described previously^17,21,22^. We made further adjustments and compared our modified aRNA protocols with the NuGEN and SMARTer protocols with nucleic acid material extracted from single cells using patch clamp technique.

## Results

In order to compare the efficiency of the different protocols, we used a standard input of 10 pg (similar to the amount in single cells) of Universal Human Reference RNA (UHR, Agilent). Patch clamp collection of cells does not yield identical amounts of RNA^4^; in fact, collection is usually incomplete, as RNA in the nucleus and in the branches of neurons may not be collected. Therefore, we also tested robustness of amplification using only 5 pg of input RNA.

Following the evaluations using UHR standards, we performed analysis of RNA collected from actual patch clamp experiments. We also performed comparison of our UHR and single cell data with publicly available data: transcriptomes for UHR standards obtained by using the original *in vitro* transcription protocol and SMARTer protocol^19^ and single cell data transcriptomes collected from embryonic brain neurons using Fluidigm C1 which utilizes the SMARTer method^23^. We, in addition, performed the original *in vitro* transcription protocol and SMARTer protocol side by side with our modified aRNA protocol (Fig. S1).

We used 5 metrics to assess the RNA-Seq data of amplified products. The first two metrics were ***total mapping rate*** defined as a fraction of raw reads mapped to the genome and transcriptome (GenCode v22, GRCh38.p2) and ***transcriptome mapping ratio*** calculated as a percentage of mapped reads which mapped to the transcriptome, excluding both rRNA (ribosomal RNA) and mtRNA (mitochondrial RNA). The third metric was ***gene model discovery rate***, assessed as the number of genes with more than 5 mapped reads detected per 3 million mapped reads. A Recent single cell RNA-Seq (scRNA-Seq) study indicated that more than one million reads are required to analyze the variance in expression^2^. We used 3 million as our baseline for our analysis to increase the gene complexity because we are targeting highly complex neuronal cells^24^.

The other two metrics, which were applied only to UHR data, assess ***reproducibility****,* measured as the Pearson coefficient of correlation of expression profiles between technical replicates, and ***accuracy****,* measured as a correlation between gene expression measured in 10 pg or 5 pg samples as compared to bulk RNA-Seq of UHR RNA. Prior to assessing reproducibility and accuracy metrics the samples were normalized by downsampling to 3 million mapped reads.

### Modification of aRNA method

**Figure 1:**
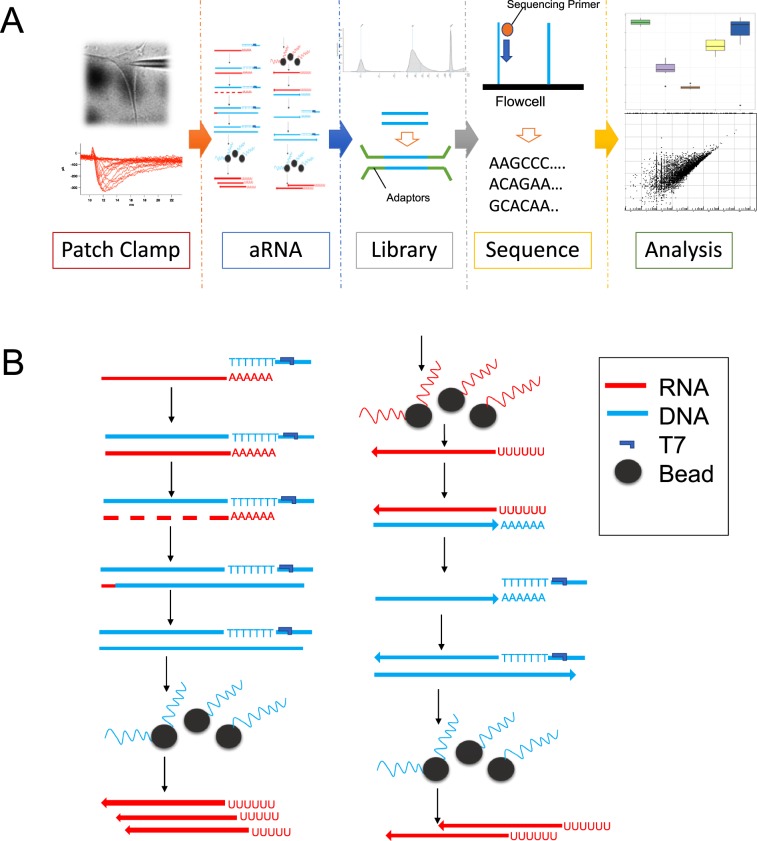
(**A**) Workflow of single cell RNAseq. After collection of cellular contents using patch pipet, RNA was amplified using aRNA amplification, and libraries were generated using Hamilton STARlet liquid handling robot following the protocol recommended by Illumina (**B**) Basic schematics of modified aRNA amplification protocol. Please see Supplemental Fig. S2 for the detailed description about the modified aRNA amplification protocol.

The most recent iteration of the aRNA protocol^3,22^ was developed for single cell applications and consists of 3 rounds of linear amplification cycles. The protocol employs column purification of nucleic acids (cDNA or aRNA at different stages of the protocol), and RNA ethanol precipitation between the cycles. Column purification and alcohol precipitation are prone to loss of nucleic acid^25,26^, which could be especially detrimental when the initial amount of material is minute. To address this potential issue, we replaced column purification of nucleic acids and ethanol precipitation with magnetic bead purification (Figs. 1B and S2). Compared to the column-based methods, purification using magnetic beads increases the yield of nucleic acids^27^ and allows elution in small volumes thus making ethanol precipitation unnecessary.

We also reduced reaction volumes at specific stages of the protocol (See Methods), which increases effective concentration of template, resulting in higher yield (Fig. S3). Decreasing loss of nucleic acid material and increasing efficiency of reactions allowed us to reduce number of rounds of amplification from three to two (see details in Methods). Elimination of one amplification step reduced noise and potential molecular bias, while saving time and decreasing the cost per sample by 47%.

**Figure 2:**
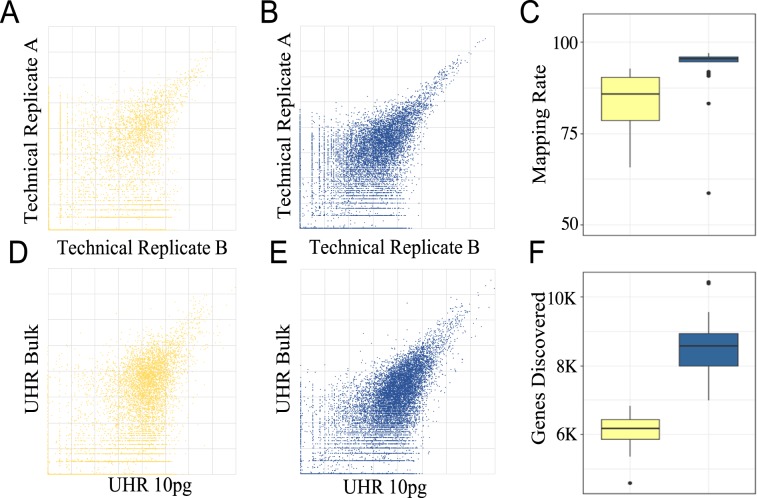
Comparison of data quality between the original^20^ and modified aRNA amplification protocols. (**A***,***B**) Correlation between technical replicates (10 pg) processed (**A**) with original and (**B**) with modified aRNA protocols. (**C**) Distribution of total mapping rates in technical replicates (10 pg) (**D,E**) Correlation between 10 pg UHR sample with 10 ng (bulk) UHR RNA processed with (**D**) original aRNA protocol and (**E**) modified aRNA protocol. (**F**) Distribution of number of genes discovered in 3,000,000 reads in samples processed with original (yellow) and modified (blue) aRNA protocols.

We sequenced 25 technical replicates of 10 pg UHR samples using the modified aRNA method and compared results with publicly available RNA-Seq data from 14 technical replicates of the same amounts of UHR processed with the original aRNA method, which used 3 rounds of amplification^19^. Samples with less than 3 million mapped reads were removed from the analysis and the remaining samples were downsampled to 3 million mapped reads to normalize the data, increasing consistency. Data quality was evaluated using the five metrics described above. A comparison indicates that the modifications we introduced to the aRNA method substantially increased data quality in every metric for 10 pg input material (Fig. 2, Tables 1, 2). This increase in data quality was even greater when the amount of UHR input RNA was decreased to 5 pg (Table 2).

**Table 1:** Comparison of 5 metrics averages of quality of scRNAseq using 10 pg of UHR.

Methods	Total Mapping Rate %	Transcriptomic Mapping Ratio %	Average number of genes detected*	Correlation between Technical Replicates*	Correlation to Bulk RNA* (Accuracy)
Original aRNA (Eberwine)	82.0	72.4	6223	0.69	0.68
Modified aRNA	92.6	78.5	8532	0.76	0.76
NuGEN	79.9	58.9	7779	0.73	0.76
SMARTer	80.0	51.1	7509	0.78	0.76

*Downsampled to 3,000,000 mapped reads.

**Table 2:** Comparison of 5 metrics averages of quality of scRNAseq using 5 pg of UHR.

	Total Mapping Rate %	Transcriptomic Mapping Ratio %	Average number of genes detected*	Correlation between Technical Replicates* (Reproducibility)	Correlation to Bulk RNA* (Accuracy)
5 pg-Original aRNA	85.6	67.1%	2452	0.34	0.42
5 pg-Modified aRNA	91.7	76.0%	4479	0.51	0.57

### Comparison of modified aRNA method with NuGEN and SMARTer protocols

The modified aRNA method showed better mapping rates, transcriptome mapping ratios and library complexities in comparison with the NuGEN and SMARTer protocols (Table 1). A reduced mapping rate for the NuGEN protocol was due to primer dimers (Table 2), suggesting self-amplification of the oligonucleotide primers when concentration of template is low. Indeed, further reduction of initial RNA quantity to 5 pg resulted in only spurious amplified primer product.

**Figure 3:**
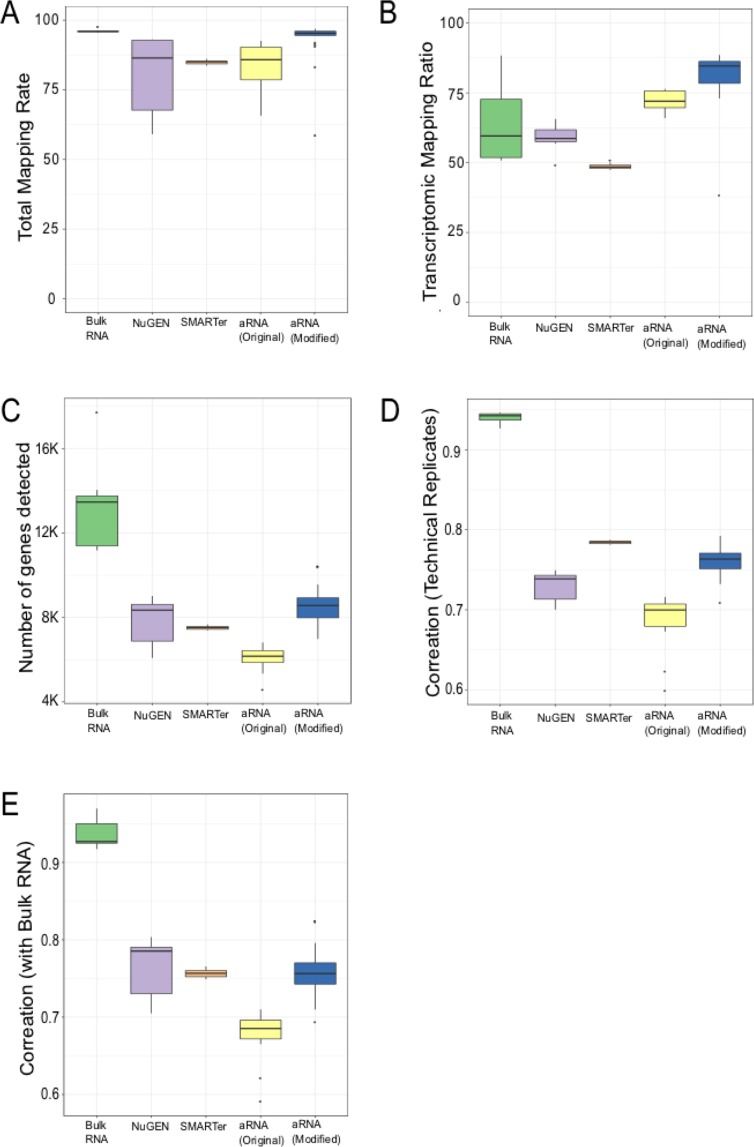
Comparison UHR samples processed using different methods (**A**) Total Mapping Rate, (**B)** Transcriptomic Mapping Ratio, (**C**) Number of Genes Discovered, (**D**) Correlation between technical replicates (Reproducibility), (**E**) Correlation with bulk RNA (Accuracy).

All three methods demonstrated similar correlation with bulk RNA (accuracy), and the reproducibility of the modified aRNA protocol was practically identical with the SMARTer protocol (0.76 vs 0.78) (Fig. 3). However, reproducibility of the SMARTer protocol may be inflated because experiments were performed in a single large batch^19^, while the aRNA protocol was performed in multiple batches, each using different tube of diluted UHR RNA. These results suggest that the modified aRNA protocol works robustly on small amounts of RNA and is suitable for single cell transcriptome studies.

### Single cell RNA-Seq

We used patch clamp to collect cytoplasmic material from 1,013 single cells from different layers of embryonic brain (Fig. S4). We then processed these cells using (a) the modified aRNA protocol followed by standard Illumina TruSeq® Stranded mRNA Library Prep (Illumina, #20020594), (680 single cells) or (b) the optimized NuGEN Ovation® RNA-Seq System V2^19^ protocol for single cells followed by NuGEN Ovation® Rapid Library Systems (NuGEN, #7102), (333 single cells). We also processed cells using original aRNA protocol but only few cells (5 cells) successfully generated single cell libraries. We compared our single cell RNA-Seq data from these methods.

To expand the comparison, we also accessed publicly available raw data (FASTQ files) from 466 single cells dissociated from fetal and adult brain^23^ (GSE67835) processed by the Fluidigm C1 instrument utilizing the SMARTer protocol followed by the Nextera library preparation. We processed raw data from all three methods (NuGEN, SMARTer, aRNA) through the same analytical pipeline in Partek Flow. Again, we downsampled each sample to 3 million mapped reads, after removal of those with fewer reads.

Amounts of RNA extracted from single cells by patch clamp can be highly variable resulting in high variation in amount of produced aRNA. We excluded samples (n = 135) that were failed to yield aRNA compared to negative control (water), which were run in parallel. In addition, there were a few outliers with an abnormally high library concentration (>100 ng/µl) (n = 2). We excluded these libraries for analyses as they had a large amount of dimer sequences and these failed the QC due to low mapping rate. Lastly, samples (n = 14) producing library amount lower than 200 ng (the minimum amount required for sequencing by Illumina protocol), were also excluded.

Single cell RNA-Seq data were analyzed using following metrics: ***total mapping rate***, ***transcriptomic mapping ratio*** and ***gene model discovery rate***. The ***total mapping rate*** was similar between SMARTer (62%) and modified aRNA methods (63%) and much lower with the NuGEN approach (24%), as a result of spurious amplification (as seen in UHR samples). The ***Transcriptomic mapping ratio*** was similar with SMARTer and modified aRNA protocols (62%), whereas it was lower with the NuGEN protocol (42%) (Table 3). The ***gene model discovery rate*** was the highest in the modified aRNA method (average number of detected genes is 3,038). The SMARTer and original aRNA approaches detected fewer genes (2,500 and 2,707, respectively), and NuGEN detected the lowest number of genes (608).

**Table 3:** Comparison of average quality metrics between RNA from embryonic brain (single cell).

Sample Names	Total Mapping Rate %	Transcriptomic Mapping Ratio %	Number of genes detected*	Sample Counts
Modified	63.02%	62.5%	3039	393
NuGEN	24.36%	42.3%	608	7
SMARTer	62.08%	62.5%	2499	442

*Downsampled to 3,000,000 mapped reads.

## Discussion

Using patch clamp to extract single cell material for RNA sequencing offers multiple advantages over approaches that involve tissue dissociation to isolate single cells. Patch clamp provides the opportunity to record location, morphology and electrophysiology of a single cell for correlation with the expression profile of the same cell. Patch clamp can be used for recording *in vivo* or in relatively intact tissue slices, obviating the need to disperse tissues to separate cells, which previous work has shown can alter gene expression.

In order to work with the minute amount of extracted single cell RNA, which often is less than the total cellular RNA content, we improved the efficiency of the procedures for RNA collection, handling and amplification. The protocols for patch clamp and collection of cytoplasm are critical, as they determine the amount of input RNA and how well it is preserved. For instance, we sliced brain samples under HEPA-filtered air hood to eliminate contamination of material during collection of cytoplasm from single cells. Additional precautions included using an RNase inhibitor in our intracellular solution, and flash-freezing the collected cytoplasm in lysis buffer in liquid LN2, immediately after mixing, to reduce potential RNA degradation.

Using reference RNA, we compared RNA sequence quality across protocols that variously employ PCR amplification, linear DNA amplification and linear RNA amplification. These protocols were then evaluated using measures of gene model discovery rate, precision, accuracy and resistance to amplification of parasitic, non-transcriptomic fragments. In our hands, linear RNA amplification based on the modified aRNA method produced the most robust results, was the most sensitive, and consistently produced higher quality data in comparison to PCR amplification.

We then tested the methods on RNA extracted from single neuronal cells from fetal human brain and compared our results with publicly available single cell RNA-Seq data. These analyses found that methods based on either linear amplification or PCR are sensitive enough and produce consistently meaningful results with single cell material. When data quality was compared, however, it becomes clear that the modified aRNA method, based on IVT, has a higher mapping rate, transcriptome ratio and gene model discovery rate, than the PCR method, confirming conclusions from comparison of these methods using UHR.

Reducing the number of rounds of RNA amplification may be a key factor contributing to the improved performance of the modified aRNA method. Every additional round requires RNA purification, cDNA synthesis and aRNA amplification, and every step is prone to variable loss of nucleic acids, increasing noise and bias. Recognizing this problem, Hashimshony *et al*.^8,18^ developed a method utilizing only one aRNA amplification cycle, by taking advantage of the increased initial cDNA amount due to multiplexing single cell samples prior to aRNA amplification. Further incremental improvements in the methodology, such as high-yield RNA transcriptases (e.g., NEB HiScribe T7 *In Vitro* Transcription kit) or sequencing library methods that require less input cDNA (e.g., Swift 2 S Library kits) may result in decreased number of linear amplification rounds to one, even without multiplexing.

## Methods

### Reference RNA

The universal human reference RNA (UHR, 740000) was purchased and dissolved in RNase-free water (Ambion, AM9932), according to manufacturer’s recommendations to 1 µg/µl stock solution. Working solutions with concentration 10 pg/µl were made by serial 1:10 dilutions and kept in low-binding microcentrifuge tubes at −80 degrees not more than two weeks.

### Patch clamping and single cell extraction

Deidentified tissues from adult human brains were acquired from medical waste after written informed consent was received from adults undergoing neurosurgery. Deidentified human fetal tissue was obtained following informed consent from parents, from elective terminations. Collection of this tissue was performed under approval by the Institutional Review Boards of both Children’s Hospital Los Angeles and the Keck School of Medicine of the University of Southern California. University of Southern California institutional review board approvals HS-12-00474 and HS-13-00399 were obtained for this work. All methods were performed in accordance with the relevant guidelines and regulations.

The embryonic brains from second trimester were sliced in a HEPA filter protected fume hood with VT1000 vibratome (Leica) into 400um slices. Neurons in tissue slices from resected embryonic brain were visually identified under illumination with an infrared Dodt gradient contrast system (homebuild). Patch pipettes (6–10 MOhm; 1.2 mm O.D.) were filled with intracellular solution (K-gluconate 130 mM; KCl 2 mM; CaCl^2^ 1 mM; MgATP 4 mM; GTP 0.3 mM; phosphocreatine 8 mM; HEPES 10 mM; EGTA 11 mM; pH 7.25 and 300 mOsm) containing recombinant RNase inhibitor (Clontech) (0.4 U/µl); and mounted in a standard patch pipette holder, connected to an automatic pressure control unit (ez-gSEAL 100b, Neobiosystem, USA). Under microscopic visualization, the patch pipette tip was maneuvered to the vicinity of a neuron, while positive pipette pressure (25–50 mmHg) was maintained. Once the pipette touched the cell membrane, gentle suction (−15 to −30 mmHg) was applied to enable formation of a giga-seal. We made the whole-cell configuration (making a seal of patch clamp pipette rim with cell membrane, and opening connection between cytoplasm and internal channel of pipette) by application of strong negative pressure to remove small patch of membrane. The content was expelled into a PCR tube containing 5 µl of lysis buffer (NaCl 350 mg, Triton 500 µl, NP-40 500 µl, deoxy 2.5 ml, Tris HCl pH 8.8 1 ml, Tris HCl pH 6.8 1.5 ml, HEPES 240 mg, pH adjusted to 8) by breaking the end of pipette tip and applying positive pressure (25–50 mmHg). The cellular material was centrifuged followed by 1 or 2 freeze thaw cycles to assure complete lysis of the material. For every cell we recorded metadata containing information about morphology, electrophysiological recording, location in tissue, details of the method used for RNA processing and library preparation along with details about batch of processing.

### Modified aRNA protocol

UHR samples (5 or 10 pg) were diluted in water to final volume of 5 µl. Alternatively, a whole volume of single cell lysate (5 µl) was used as a starting material for the protocol.

### First round of aRNA amplification

#### First strand synthesis

To the 5 ul of each sample, we added: 4.9 µl of RNase free water, 2.4 µl of 5x First Strand buffer (Life Technology, included with Superscript III), 1.2 µl of dNTPs (Thermo Fisher, R0193, 10 mM), 0.45 µl of 100 mM DTT (Life Technology, included with Superscript III) and 0.3 µl of dt-T7 oligo (Invitrogen, GGAGGCCGGAGAATTGTAATACGACTCACTATAGGGAGACGCGTGTTTTTTTTTTTTTTTTTTTTTTTTV, 10 ng/µl). This entire mixture was incubated for 5 minutes at 70 degrees to denature the RNA, then immediately placed on ice and the first strand enzyme mix was added: 0.3 µl of Rnasin (Promega, N2111, 2500 unit), 0.45 µl of Superscript III (Life Technology, 18080044), 1 µl of RNAse-free water. First strand synthesis was performed at 42 °C for 30 minutes, followed by 70 °C for 15 minutes.

#### Second strand synthesis

To the 9.35 ul of the above reaction, we added second strand mix (5.56 µl of 5X Second strand buffer (Life Technology, 10812014), 0.75 µl of dNTP mix, 1 µl of DNA polymerase I (10 U/µl, Life Technology, 18010017), 0.25 µl of RNase H (2 U/µl, Life Technology, 18021071), 8.26 µl of RNase-free water) and incubated for exactly 2 hours at 16 °C. After incubation, 1 µl of T4 DNA polymerase (5 U/µl, Life Technology, 18005025) was added and samples incubated for an additional 10 minutes at 16 °C. Double stranded DNA was then purified using 52 µl of Agencourt XP RNAclean beads (Agencourt, A63987) according to manufacturer’s protocol and eluted in 4 µl of water.

#### *In vitro* transcription

Reagents from Ambion MEGAscript® T7 Transcription Kit (Life Technology, AMB13345) were used for aRNA amplification. The 1 µl of each ATP, GTP, CTP, TTP, 10X reaction buffer, 10x Enzyme mix were added to each sample followed by incubation at 37 °C for 14 hours. Amplified RNA was purified with 18 µl of Agencourt RNACleanXP Beads and eluted in 4 µl of water.

### Second round of aRNA amplification

#### First strand synthesis

After addition of 1 µl of Random primers (0.05 µg/µl, Promega, PAC1181), samples were incubated at 70 °C for 10 minutes. First strand synthesis was performed by adding first strand mastermix (2 μl of 5x First Strand buffer, 0.5 μl of dNTP mix, 0.5 μl of RNasin, 1 μl of DTT (100 mM), 1 μl of Superscript III) and successive incubations at RT (25 °C) for 10 minutes, 42 °C for 30 minutes, 95 °C for 5 minutes.

#### Second strand synthesis

1 µl of dt-T7 oligo (10 ng/µl) was added to the above 10 ul first strand reaction for each sample. After 5 min of incubation at 70 °C, second strand master mix was added (7.5 µl of 5X Second strand buffer, 0.75 µl of dNTPs, 1 µl of DNA polymerase I, 17.25 µl of water) and samples were incubated exactly 2 hours at 16 °C. After incubation, 1 µl of T4 DNA polymerase (5 U/µl, Life Technology, 18005025) was added and samples incubated for an additional 10 minutes at 16 °C. Samples were then immediately purified with 70 µl of Agencourt RNACleanXP Beads and eluted in 4 µl of water.

#### *In vitro* transcription

*In vitro* transcription was performed the same way as in the first round, and samples were purified with Agencourt RNAClean XP beads. RNA samples were eluted in 50 µl of RNase-free water and the amount of aRNA was measured using Agilent RNA ScreenTape (Agilent, 5067–5576) on an Agilent 2200 Tapestation.

### Library preparation

For bulk RNA samples (100 ng of UHR) and 10 pg UHR samples processed with modified aRNA protocol libraries were generated using Illumina Truseq Stranded mRNA Library Preparation kit (Illumina, RS-122-2101 and 2102). Samples from aRNA (round 2) were dried with SpeedVac (Savant, SVC100D) into low volume (less than 1ul) and added to the Elute-Prime-2 stage of the TruSeq Stranded mRNA protocol (Illumina, 20020594) to bypass the poly-A mRNA selection process. Rest of the steps were performed, following the manufacturer’s protocol using TruSeq RNA Single Indexes Set A and B (Illumina, 20020492 and 20020493).

For samples processed with Ovation® RNA-Seq System V2 (NuGEN, 7102-32), sequencing libraries were made using NuGEN Encore Rapid Library system (NuGEN, 0319, 0320) according to the protocol previously reported^19^.

### Sequencing and data analysis

Size distribution of sequencing libraries was assessed by Agilent D1000 Screen Tape (5067-5582) on an Agilent 2200 Tapestation. Library concentrations were measured by KAPA Library Quantification Kits for Illumina® Platforms (KAPA, KK4828-07960166001). 24 libraries were multiplexed and sequenced on an Illumina HiSeq 2500 System on Rapid flowcells (GD-402-4001) using HiSeq Rapid SBS Kit V2 with 100 bp single-ended reads.

Sequencing reads were trimmed from both ends based on quality score using filter module in Partek Flow with settings of 25 minimum read length. The trimmed reads were mapped to human genome (hg38) and transcriptome (GENCODE Genes - release 26) using STAR aligner (STAR - 2.5.3a) within Partek Flow pipeline (Max junctions: 1,000,000, Max mismatches: 10, Max seeds per read: 1000). Total mapping rate was measured as part of standard Post Alignment QA/QC modules in Partek Flow. Read counts were quantified for each sample using STAR transcript/gene expression quantification function, and the transcriptome mapping ratio was taken from Quantification summary. For further analysis BAM files were downsampled to 3,000,000 mapped reads using the SAMtools module in Partek Flow. Samples with less than 3,000,000 mapped reads were excluded. The reads were assigned to genes using htseq module within Partek Flow. In addition, number of reads was normalized with quantile normalization prior to comparison. For every sample we calculated the number of genes detected per 3,000,000 mapped reads (defined as genes with more than 5 uniquely mapped reads), Pearson coefficients of correlation between gene expression of technical replicates and between each sample and bulk RNA. For comparison with in-house SMARTer and original aRNA protocol, we compared the gene discovery rate with samples downsampled to 100,000 reads as there was limited number of reads on these samples.

## Supplementary information

Supplementary Information.

## Data Availability

Datasets are accessible in GSE144216.
