# Supplementary material for: Robust RNA-Seq of aRNA-amplified single cell material collected by patch clamp
Source: Sci Rep. 2020 Feb 6;10:1979. doi: 10.1038/s41598-020-58715-y (PMC7004989; doi:10.1038/s41598-020-58715-y)
Supplement: Supplementary file 1 — Supplementary Information. [file 41598_2020_58715_MOESM1_ESM.pdf]

Robust RNA-Seq of aRNA-amplified single cell material collected by patch clamp

Authors and Affiliations

Jae Mun "Hugo" Kim[1,3,*], Adrian Camarena[1,4], Christopher Walker[1], Ming Yi Lin[1], Victoria Wolseley[1], Tade Souaiaia[1,2], Matthew Thornton[1], Brendan Grubbs[1], Robert H. Chow[1], Oleg V Evgrafov[1,2] and James A Knowles[1,2,*]

1. Zhilka Neurogenetic institute, University of Southern California.
   1501 San Pablo St, Los Angeles, CA 90033
2. SUNY Downstate Medical Center
   450 Clarkson Ave, Brooklyn, NY 11203
3. University of California, San Diego
   9500 Gilman Dr, La Jolla, CA, 92093
4. University of Chicago, Pritzker School of Medicine
   924 E 57th St Suite 104, Chicago, IL, 60637

* Corresponding authors: Jae Mun "Hugo" Kim (jmk002@health.ucsd.edu) and James A Knowles (James.knowles@downstate.edu)

Supplementary information

Supplemental Figure legends

Figure S1. Comparison UHR samples processed using different methods (compared side by side in house). A number of genes discovered was compared with reads downsampled to 100,000.

Figure S2. Detailed protocol diagram of modified aRNA protocols.

Figure S3. Amplified aRNA amount comparison between 3-round original volume and half volume aRNA amplification from 5pg of UHR. The amounts were indicated by ng.

Figure S4. Different layers where the single cells were collected from. We collected several Cajal Retzius cells, multiple layers of Cortical plate, few layers of Subplate, ventricular zone and sub ventricular zone. Scale Bar=10um

Supplementary Figures

Figure S1

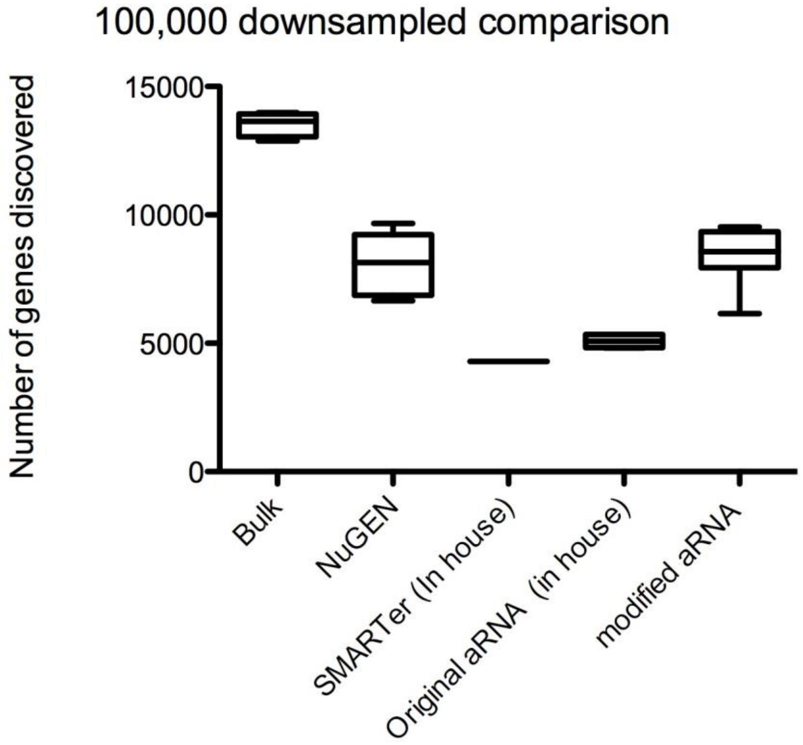

Figure S2

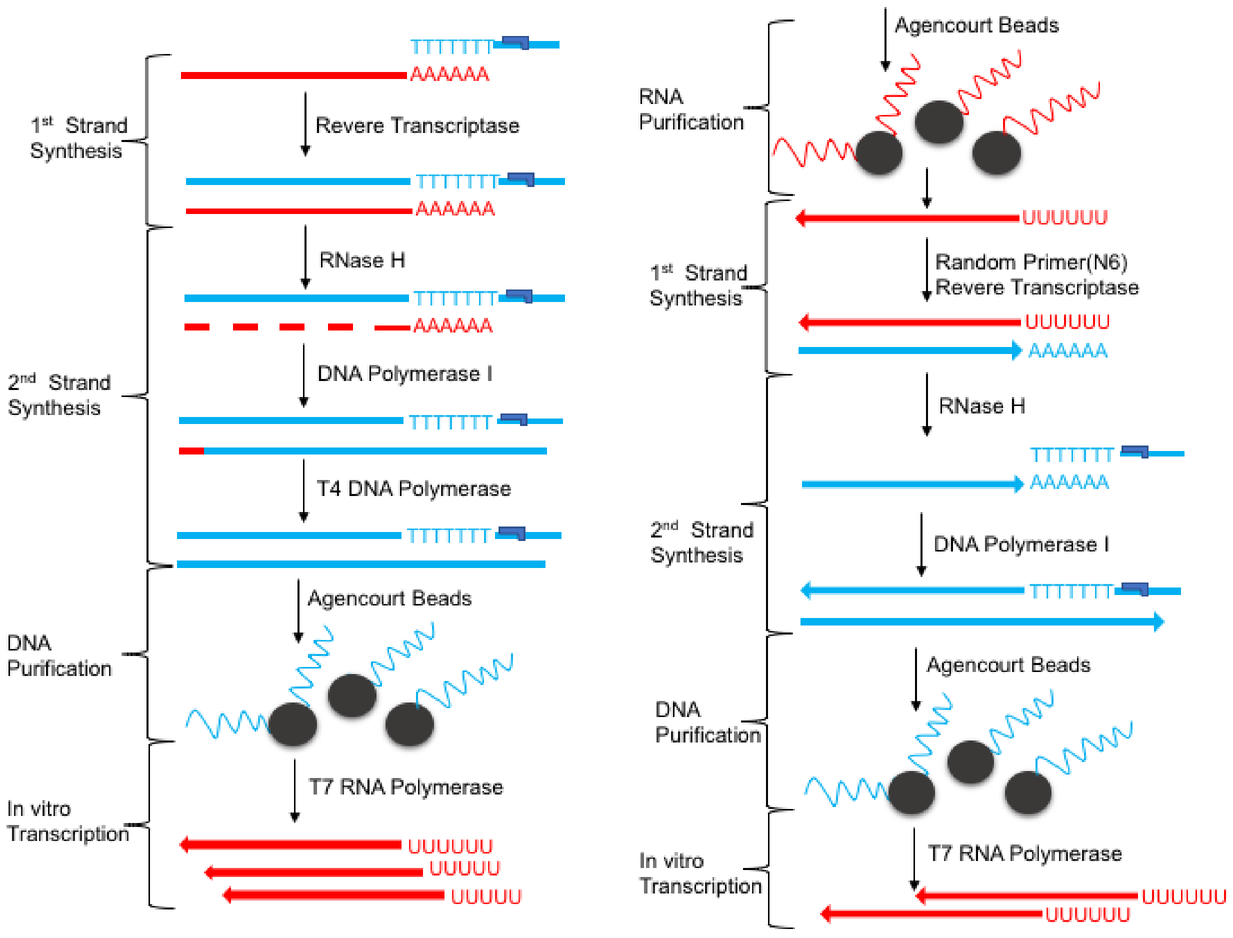

1st Strand Synthesis
TTTTTTT
AAAAAA
Revere Transcriptase
2nd Strand Synthesis
RNase H
DNA Polymerase I
T4 DNA Polymerase
DNA Purification
Agencourt Beads
In vitro Transcription
T7 RNA Polymerase
UUUUUU
UUUUU
RNA Purification
Random Primer(N6)

Figure S3

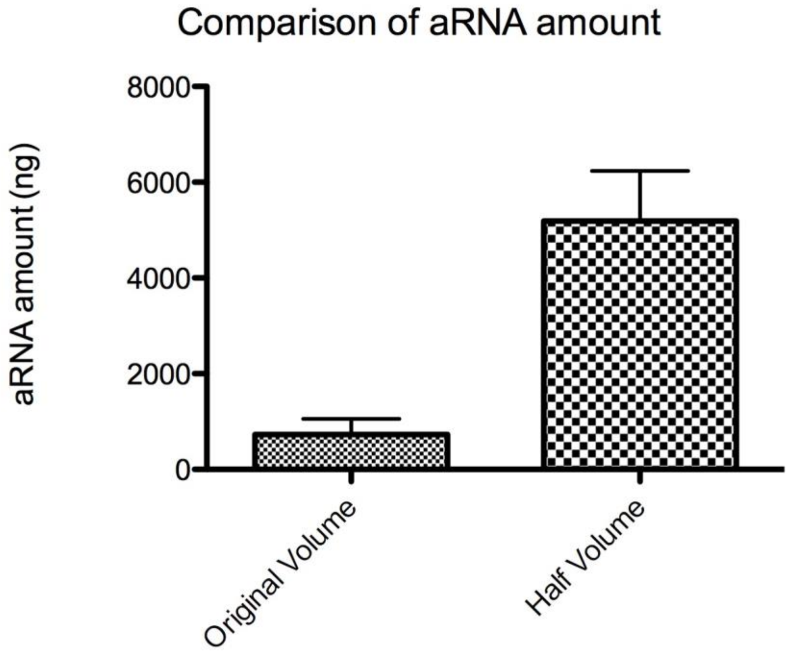
Comparison of aRNA amount
aRNA amount (ng)
8000
6000
4000
2000
0
Original Volume
Half Volume

Figure S4

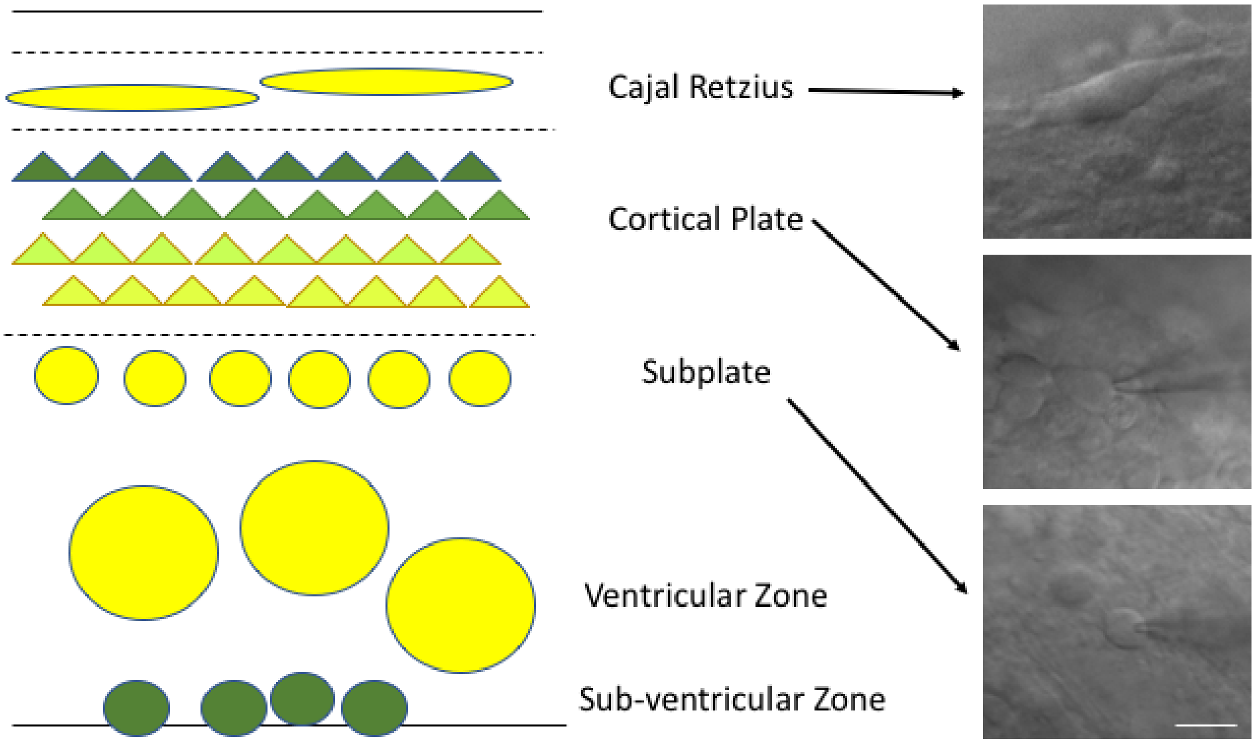

Cajal Retzius
Cortical Plate
Subplate
Ventricular Zone
Sub-ventricular Zone
